# Supplementary material for: The Scm FCS finger binds the Pcl Tudor domain to link canonical PRC1 and PRC2.1 in Drosophila
Source: Protein Sci. 2026 Nov 9;35(12):e70855. doi: 10.1002/pro.70855 (PMC13746325; doi:10.1002/pro.70855)
Supplement: Supplementary file 1 [file PRO-35-e70855-s001.docx]

**TABLE S1**

| Sample | Expected mass (Da) | Identified mass (Da) | Match error | Label |
| --- | --- | --- | --- | --- |
| Scm_FCS_  +  Pcl_Tudor_ | 7809.7 | 7809.794 | 0.094 | Pcl_Tudor_ |
|  | 9654.02 | 9653.014 | -1.006 | 2[Zn] Scm_FCS_ |
|  | 17400.36 | 17400.565 | 0.205 | 1[Zn] Scm_FCS_ + Pcl_Tudor_ |
|  | 17463.72 | 17463.608 | -0.112 | 2[Zn] Scm_FCS_ + Pcl_Tudor_ |
| Scm_FCS-R96D_  _+_  Pcl_Tudor_ | 7809.7 | 7809.616 | -0.084 | Pcl_Tudor_ |
|  | 9612.72 | 9612.556 | -0.164 | 2[Zn] Scm_FCS-R96D_ |
| Scm_FCS_  +  Pcl_Tudor-D384R_ | 7850.9 | 7850.842 | -0.058 | Pcl_Tudor-D384R_ |
|  | 9654.02 | 9653.354 | -0.666 | 2[Zn] Scm_FCS_ |
| Scm_FCS-R96D_  +  Pcl_Tudor-D384R_ | 7850.9 | 7850.947 | 0.047 | Pcl_Tudor-D384R_ |
|  | 9549.36 | 9549.34 | -0.020 | 1[Zn] Scm_FCS-R96D_ |
|  | 9612.72 | 9612.679 | -0.041 | 2[Zn] Scm_FCS-R96D_ |
|  | 17463.62 | 17465.987 | 2.367 | 2[Zn] Scm_FCS-R96D_ + Pcl_Tudor-D384R_ |
